# Consumption of Traditional Fruits and Vegetables among Children in the US-Affiliated Pacific Region

**DOI:** 10.1093/cdn/nzac101

**Published:** 2022-06-16

**Authors:** Rica Dela Cruz, Eric Wolfe, Kim M Yonemori, Marie K Fialkowski, Lynne R Wilkens, Patricia Coleman, Sunema Lameko-Mua, Emihner Johnson, Daisy Gilmatam, Cecilia Sigrah, Moria Shomour, Shelley Remengesau, Julia Alfred, Mark Acosta, Reynolette Ettienne, Jonathan Deenik, Tanisha F Aflague, Randall Nelson, Kristina Abello Salazar, Rachel Novotny, Carol J Boushey

**Affiliations:** University of Hawaii Cancer Center, Honolulu, Hawaii; University of Hawaii Cancer Center, Honolulu, Hawaii; University of Hawaii Cancer Center, Honolulu, Hawaii; University of Hawaii, College of Tropical Agriculture and Human Resources, Honolulu, Hawaii; University of Hawaii Cancer Center, Honolulu, Hawaii; Northern Marianas College, Saipan, Northern Mariana Islands; American Samoa Community College, Pago Pago, American Samoa; Island Food Community of Pohnpei, Pohnpei, Federated States of Micronesia; Yap Health Services, Yap, Federated States of Micronesia; Kosrae Community Health Center, Kosrae, Federated States of Micronesia; Chuuk State Division of Public Health, Federated States of Micronesia; Palau Community College, Koror, Republic of Palau; Ministry of Health, Majuro, Republic of the Marshall Islands; University of Guam, Mangilao, Guam; University of Texas at San Antonio, San Antonio, TX, USA; University of Hawaii, College of Tropical Agriculture and Human Resources, Honolulu, Hawaii; University of Guam, Mangilao, Guam; Northern Marianas College, Saipan, Northern Mariana Islands; University of Hawaii Cancer Center, Honolulu, Hawaii; University of Hawaii, College of Tropical Agriculture and Human Resources, Honolulu, Hawaii; University of Hawaii Cancer Center, Honolulu, Hawaii

**Keywords:** nutrition, diet, food, fruit, vegetable, child, Pacific Islands, Indigenous peoples

## Abstract

**Background:**

Traditional Pacific diets have many health benefits, including maintenance of a healthy weight and prevention of various diseases. Few studies have evaluated the frequency at which traditional diets are consumed in the Pacific, especially among children.

**Objectives:**

This study examined the frequency of traditional and acculturated fruit and vegetable (F&V) intake among children in the US-affiliated Pacific (USAP) region.

**Methods:**

Diet records of 3319 children ages 2 to 8 y old were analyzed for frequency of traditional or acculturated F&V intake within USAP jurisdictions of American Samoa, Commonwealth of the Northern Mariana Islands (CNMI), Federated States of Micronesia (FSM; FSM island states include Chuuk, Kosrae, Pohnpei, and Yap), Guam, Hawaii, Republic of the Marshall Islands (RMI), and Republic of Palau.

**Results:**

Of the 95,304 food items recorded among participating children in the USAP jurisdictions, 15.2% were F&Vs. Of the 10 jurisdictions, children in the islands of Chuuk, Kosrae, Yap, and Pohnpei recorded the highest frequencies of traditional F&V intake relative to their total F&V intake (67.8%, 64.8%, 56.7%, and 52.5%, respectively). American Samoa and RMI recorded moderate frequency of traditional F&V intake (38.9% and 46.4%, respectively), whereas children in Hawaii, Guam, and CNMI recorded the lowest frequencies of traditional F&V intake relative to their total F&V intake (10.4%, 12.4%, and 15.3%, respectively). Children in Hawaii, Guam, Palau, and CNMI recorded high frequencies of acculturated F&V intake (37.8%, 31.2%, 34.5%, and 27.9%, respectively).

**Conclusions:**

Overall, children in the USAP jurisdictions participating in this study recorded a low frequency of F&V intake. The differences in traditional F&V intake found between the USAP islands may be due to variation in economic income level and external influences on social and cultural norms among the island populations and variations of cost, accessibility, and convenience of each category of food to each island's population.

## Teaser

Among 3319 children, aged 2–8 y, residing in 10 jurisdictions of the US-affiliated Pacific region, only 27.8% of the fruits and vegetables eaten were considered traditional foods.

## Introduction

Indigenous peoples around the world have experienced dramatic shifts in dietary patterns as a result of historical colonization and modern globalization (1–4). One region that has seen a rapid nutrition transition is the Pacific (2–4). Prior to Western contact, Indigenous peoples of the Pacific depended on their own self-sustaining, local food systems, consuming only what came from the land and sea around them (4). However, foreign colonization and even increasing interaction with other global cultures has led to the introduction of many new foods and to new methods of preparing food into the Pacific Islander diet (4). Today, a variety of foods, including fresh produce and processed foods, such as canned goods, are imported across Oceania, including the US-affiliated Pacific Islands (USAPI) (5, 6).

The movement away from traditional diets based on locally grown foods and a shift towards acculturated foods characterized by introduced and imported foods is considered one of the contributing factors that has led to adverse effects on dietary quality and health among Indigenous Pacific populations (4, 5). Other factors to be considered in this move away from traditional diets is a parallel movement away from the traditional model of a subsistence-based economy found in many of the Pacific Island communities prior to the contact with Western cultures, where much of the effort and activity to these communities were focused on the production of local foods as a matter of survival.

According to the WHO, 8 of the world's 10 most obese nations are Pacific Island Countries or Territories (PICTs), with at least one-third of all adults in the region living with overweight or obesity in 2014 (7). In both adults and children, BMI has risen at a higher rate in the Pacific compared with high-income Western countries since 1980 (8, 9). Noncommunicable diseases (NCDs; e.g., diabetes, cardiovascular disease, and cancer) among Pacific Islanders are believed to have increased due to the rise in unhealthy diets and obesity (5, 10, 11). NCDs are responsible for about 70% of all deaths in PICTs and have led to the decrease in life expectancy in many of these islands (10). Due to the alarming increase in these diseases and its burden on the health care system, in 2010 the USAPI declared a regional emergency due to the epidemic of NCDs, which still remains in effect today (12).

Several studies have found evidence to show that traditional Pacific diets provide many health benefits, can support maintenance of a healthy weight, and may prevent NCDs, such as cardiovascular disease and diabetes (13, 14). Common traditional foods of the Pacific include starchy vegetables such as breadfruit, root plants such as taro and sweet potato, leafy greens such as water spinach, fruits such as coconut and banana, and a variety of fish and other seafood. Traditional Pacific diets, in general, have been shown to be low in fat and animal products, and high in complex carbohydrates, fiber, and vegetables compared with diets of industrialized nations (14, 15). Research also shows better nutrient profiles from traditional food consumption. One study found that traditional food contributed more protein, phosphorus, iron, zinc, copper, magnesium, and vitamin A across various age groups, whereas Western foods from the market contributed to greater amounts of dry weight, energy, fat, carbohydrate, calcium, and sodium (16). A study in preschool-aged children also found that a higher intake of traditional foods resulted in higher intakes of vitamins A and D, iron, magnesium, and zinc (17). An intervention promoting traditional Pacific foods resulted in increased provitamin A carotenoid intake (18). In addition to the types of foods consumed, traditional ways of cooking or food preparation are also seen as part of traditional diets. These cooking methods include baking, steaming, and roasting over a fire, which are much healthier than the introduced method of frying in oil (13). Indeed, improved nutrition from traditional foods can positively influence health outcomes and prevent various NCDs.

Both adults and children can greatly benefit from the consumption of Pacific traditional diets. A limited number of studies have evaluated the frequency at which traditional diets are consumed in the Pacific. Even fewer have assessed this in young children (6, 14, 18–22). The purpose of this study was to assess the frequency of consumption of traditional or acculturated fruits and vegetables (F&Vs) among children in the US-affiliated Pacific (USAP) region. To our knowledge, no other studies have comprehensively assessed the frequency at which traditional versus acculturated F&Vs are consumed among children of this subregion of the Pacific. Understanding this can provide insight into how diets of children living in the USAP can be enhanced to promote good health.

## Methods

### Study Population

The Children's Healthy Living Program for Remote Underserved Minority Populations of the Pacific Region (CHL) is a partnership among universities, local organizations, and other collaborators across the USAP region. This USAP region is composed of 11 jurisdictions: the US states of Alaska and Hawaii, the US territories of American Samoa, the Commonwealth of the Northern Mariana Islands (CNMI) and Guam, and the Freely Associated States (FAS) of the Federated States of Micronesia (FSM; FSM island states of Chuuk, Kosrae, Pohnpei, and Yap were separate jurisdictions for this study), the Republic of the Marshall Islands (RMI), and the Republic of Palau (23). Alaska was excluded from this study because it is the only CHL jurisdiction that lies outside of the tropical Pacific region.

Children ages 2 to 8 y old and their parents or caregivers living in selected communities of each USAP jurisdiction were recruited to participate in the study from 2012 to 2016. Recruitment efforts occurred primarily at early childhood education centers, elementary schools, and community events (24, 25). In the FAS jurisdictions, 200 children per jurisdiction was the recruitment goal. Among the US states and territories, there was a recruitment goal of 150–180 children per selected community with evaluable anthropometry and from these children, with a goal of 100 completed dietary records per community. Four communities participated within each state or territory (26).

Institutional review board (IRB) approval was obtained from the University of Hawaii at Mānoa (Honolulu), University of Guam (Mangilao), and the Republic of Palau. All other jurisdiction institutions ceded to the University of Hawaii at Mānoa. Parental/guardian consent and child assent were obtained.

### Dietary records

Two-day dietary records, referred to as “food logs” among the participants, were completed by a surrogate (either a parent or caregiver) and were used to assess food-group intakes of the children. Surrogates were asked to complete the food log for their children on 2 randomly assigned nonconsecutive days, to ensure representation of all days of the week across children. Over each of these days, surrogates recorded the food and beverages consumed by the child, the time of eating, who prepared the food, where the food was eaten, and any activity the child was doing while eating (if any). In Palau, Yap, Chuuk, Pohnpei, Kosrae, RMI, and American Samoa, recording was done in the Indigenous language if preferred. This was then translated into the English language prior to diet data entry. Details of food logs have been described previously (25, 26). Trained staff entered the food-log data into the Pacific Tracker3 (PacTrac3) dietary and physical activity assessment program (25, 27). A systematic review process confirmed entries of the 2-d dietary records completed by surrogates.

### Food classifications

Foods were classified into 2 broad categories: “traditional” or “acculturated.” These categories were based on concepts of traditional (foods that sustained the culture a very long time ago) and acculturated (foods that possess cultural meaning and are not considered traditional but have been adopted into the everyday eating habits of many individuals) foods in the Pacific, which were derived from the literature (15, 28). In the classification process, CHL staff from participating jurisdictions, who were both Indigenous and nutrition experts in their respective jurisdictions, were tasked to complete 2 forms: *1*) a Food Identity Questionnaire that captured culturally relevant definitions of traditional and acculturated foods and *2*) a Food Classification Form, which consisted of 126 food items commonly classified as F&Vs and suggested by CHL jurisdiction staff (see **Supplemental Table 1** for list of food items enumerated). The foods listed on the Food Classification Form were taken from the PacTrac3 dietary database (25, 29) and focused on F&Vs, which aligned with 1 of CHL's goals to increase consumption of F&Vs. No specific species or variety of F&V were included in the forms and listed only the general term of each F&V (e.g., “banana,” “mango,” “eggplant”). Through the completion of these 2 forms, each Pacific Island jurisdiction determined which F&Vs would be placed in which category. Using the list of fruits and vegetables from PacTrac3 provided a common starting point for each jurisdiction in the classification process.

Using the 2 forms gathered from all of the jurisdictions, each of the 126 F&V food items were given a classification of either traditional or acculturated, based on which category received the most counts among all of the jurisdictions. F&Vs counted as traditional in certain jurisdictions and acculturated in another jurisdiction (e.g., cassava, jackfruit, and pumpkin) were classified as traditional or acculturated based on which classification had the higher count among all the jurisdictions. After completing the classification process, recorded F&Vs in the children's food logs could then be enumerated as either traditional or acculturated. For this study, only whole or single F&Vs that were included in the Food Classification Form food item list were enumerated as traditional or acculturated. Whole or single F&Vs that were not listed in the classification form but were included in dietary records as consumed by children (e.g., apples, honeydew melon, pears, and peaches) were classified as “neither traditional nor acculturated.” Mixed foods, such as “apple turnover” or “banana lumpia” for example, were not enumerated as these are not whole fruits or vegetables. These methods have been further described as part of CHL's Exploring Foods of the Pacific Study (30).

### Data analysis

The Statistical Package for the Social Sciences (SPSS) version 23 (IBM Corporation) was used to carry out all statistical analyses. This analysis includes the baseline data of dietary records collected by the CHL program. Descriptive statistics were used to analyze the frequency of F&V consumption by food classification (traditional or acculturated) and further stratified by jurisdiction. F&Vs not designated as part of the Food Classification Form food item list were categorized as “neither traditional nor acculturated” in this analysis. Percentages are presented in terms of the total frequency of F&Vs recorded among all jurisdictions as well as the frequency of F&Vs recorded within each jurisdiction.

## Results

A total of 3319 children aged 2–8 y old were included in this study (**Table 1**). Guam had the highest number of children (*n* = 687) and Chuuk had the lowest number of children (*n* = 124). The time period of data collection varied between the FAS jurisdictions and the state and territory jurisdictions. Data collection in the FAS was completed within 1 annual quarter, whereas data collection in the 4 intervention sites spanned across the better part of a year.

**TABLE 1:** Summary of Children's Healthy Living Program Pacific Island jurisdictions among children 2–8 y old^1^

Jurisdiction	Total number of unique child participants^2^	Data collection period^3^	Economic income level^4^	US affiliation status
American Samoa	610	Q1, Q2, Q3, Q4	Upper middle income	Territory
Chuuk	124	Q2	Lower middle income	Freely associated
CNMI	559	Q1, Q2, Q4	High income	Territory/Commonwealth
Guam	687	Q1, Q2, Q3, Q4	High income	Territory
Hawaii	441	Q1, Q2, Q3, Q4	High income	State
Kosrae	129	Q1	Lower middle income	Freely associated
Palau	172	Q2	Upper middle income	Freely associated
Pohnpei	206	Q4	Lower middle income	Freely associated
RMI	195	Q1	Upper middle income	Freely associated
Yap	196	Q3	Lower middle income	Freely associated

1 *n* = 3319. CNMI, Commonwealth of the Northern Mariana Islands; Q, quarter; RMI, Republic of the Marshall Islands.

2 Includes total number of individual participants across the whole data collection period (i.e., all quarters).

3 Q1 =  January to March; Q2 = April to June; Q3 = July to September; Q4 = October to December.

4 Economic income levels were defined by the 2011 World Bank classifications, which was around the time of diet data collection.

A total of 95,304 food items among all food groups were recorded as eaten by all participating children in all of the Pacific jurisdictions (**Table 2**). Of these food items, 14,484 (15.2%) were fruits or vegetables consumed by Pacific children. American Samoa children had the highest consumption of F&Vs (19.1%) followed by Yap (18.6%) and Hawaii (17.1%). Children in Chuuk had the lowest consumption of F&Vs (5.7%), and just below was RMI (7.4%).

**TABLE 2:** Recorded frequency of fruits and vegetables among CHL children by jurisdiction^1^

	Jurisdiction										
	American Samoa	Chuuk	CNMI	Guam	Hawaii	Kosrae	Palau	Pohnpei	RMI	Yap	All jurisdictions
Total food items using 2-d dietary record											
*n*	21,602	2407	16,221	19,553	14,198	2985	4183	5243	3709	5203	95,304
%	100	100	100	100	100	100	100	100	100	100	100
Fruits or Vegetables											
*n*	4124	270	2307	2630	2431	293	513	673	274	969	14,484
%	19.1	5.7	14.2	13.5	17.1	9.8	12.3	12.8	7.4	18.6	15.2
Neither Fruits nor Vegetables											
*n*	17,478	2137	13,914	16,923	11,767	2692	3670	4570	3435	4234	80,820
%	80.9	88.8	85.8	86.5	82.9	90.2	87.7	87.2	92.6	81.4	84.8

1 *n* = 3319. All food items recorded in dietary records for 2 d for each child. CHL, Children's Healthy Living Program for Remote Underserved Minority Populations of the Pacific Region; CNMI, Commonwealth of the Northern Mariana Islands; RMI, Republic of the Marshall Islands.

Of the F&Vs consumed by all children in all jurisdictions, 27.8% were classified as traditional and 25.8% were classified as acculturated, with 46.4% of the recorded F&Vs classified as “neither traditional nor acculturated” (**Table 3**).

**TABLE 3:** Frequency of traditional and/or acculturated fruits and vegetables among CHL children 2–8 y old by jurisdiction^1^

	Jurisdiction										
	American Samoa	Chuuk	CNMI	Guam	Hawaii	Kosrae	Palau	Pohnpei	RMI	Yap	All jurisdictions
Traditional or acculturated											
*n*	2330	223	997	1146	1172	249	263	464	151	763	7758
%	56.5	83.6	43.2	43.6	48.2	85.0	51.3	68.9	51.1	78.7	53.6
Neither traditional nor acculturated											
*n*	1794	47	1310	1484	1259	44	250	209	123	206	6726
%	43.5	17.4	56.8	56.4	51.8	15.0	48.7	31.1	44.9	21.3	46.4
Traditional											
*n*	1606	183	535	325	253	190	86	353	127	549	4025
%	38.9	67.8	15.3	12.4	10.4	64.8	16.8	52.5	46.4	56.7	27.8
Nontraditional											
*n*	2518	87	1954	2305	2178	103	427	320	147	420	10,459
%	61.1	32.2	84.7	87.6	89.6	35.2	83.2	47.5	53.6	43.3	72.2
Acculturated											
*n*	724	40	644	821	919	59	177	111	24	214	3733
%	17.6	14.8	27.9	31.2	37.8	20.1	34.5	16.5	8.8	22.1	25.8
Non-acculturated											
*n*	3400	230	1663	1809	1512	234	336	562	250	755	10,751
%	82.4	85.2	72.1	68.8	62.2	79.9	65.5	83.5	91.2	77.9	74.2

1 *n* = 3319. Based on total fruit or vegetables shown in Table 2. CHL, Children's Healthy Living Program for Remote Underserved Minority Populations of the Pacific Region; CNMI, Commonwealth of the Northern Mariana Islands; RMI, Republic of the Marshall Islands.

Chuuk, Kosrae, Yap, and Pohnpei children had the highest amounts of traditional F&Vs recorded as consumed within their jurisdictions at 67.8%, 64.8%, 56.7%, and 52.5%, respectively. Hawaii, Guam, and CNMI had the lowest amounts of traditional F&Vs recorded as consumed within their jurisdictions at 10.4%, 12.4%, and 15.3%, respectively.

Hawaii, Guam, Palau, and CNMI had the highest amounts of acculturated F&Vs recorded as consumed within their jurisdictions at 37.8%, 31.2%, 34.5%, and 27.9%, respectively. RMI, Chuuk, and Pohnpei had the lowest amounts of acculturated F&Vs recorded as consumed within their jurisdictions at 8.8%, 14.8%, and 16.5%, respectively.

## Discussion

F&Vs were a low proportion of the total food items consumed by children from the USAP jurisdictions. Even the jurisdiction with the highest percentage of F&V intake still recorded less than one-quarter fruits or vegetables being part of the overall children's food logs. These results are consistent with the CHL intervention study, limited to 5 USAPI jurisdictions (Alaska, American Samoa, CNMI, Guam, and Hawaii) with a focus on increasing access and intakes of healthy foods such as F&Vs (26). The results of dietary records analyzed after the community intervention showed no significant increase in F&V intake (31).

Despite some jurisdictions having low records of F&V intake, these islands are consuming a high percentage of traditional F&Vs among those consumed. Children from Chuuk had the least F&V intake of all jurisdictions but had the highest percentage of traditional F&Vs recorded within their jurisdiction. Similarly, Kosrae and Pohnpei had relatively lower intakes of F&Vs compared with other jurisdictions, but also had a higher percentage of traditional F&Vs recorded within their jurisdictions.

The jurisdictions with the highest percentages of recorded traditional F&Vs (FSM island states: Chuuk, Kosrae, Yap, and Pohnpei) are those classified at lower-middle-income economic levels, as reported by the World Bank at the time of data collection (32) (Table 1 presents the 2011 economic income levels of each jurisdiction). Lower-middle-income economies were those with a gross national income (GNI) per capita, between $1026 and $4035; upper-middle-income economies were those with a GNI per capita between $4036 and $12,475; high-income economies were those with a GNI per capita of $12,476 or more (33). In contrast, the Pacific jurisdictions that recorded less traditional and more acculturated F&V intake (CNMI, Guam, and Hawaii) are those classified at high-income economic levels (32).

Much of this difference in traditional F&V intake may be explained by the differences in availability (i.e., amount) and accessibility (i.e., cost) of imported foods. High-economic-income-level jurisdictions are more developed and have better infrastructure that allows for a greater means to import goods, including food. As a result, these acculturated imported foods are more available and accessible in places such as Hawaii and Guam, presumably causing people to shift away from producing, purchasing, and/or consuming traditional, locally grown foods (1). On the other hand, lower-middle-economic-income jurisdictions with less of a global commerce infrastructure, such as the island states of FSM, do not have the same availability to a wide range of imported goods. Foods that are imported to these jurisdictions are also more costly compared with places where imported goods are readily available, and more costly compared with traditional foods, which may benefit from a local barter system or a community sharing mentality (34, 35). They are also likely of poor quality due to less frequent shipping schedules (36). Thus, people may be less likely to purchase and consume acculturated imported fruits and vegetables and are more likely to continue practicing and interfacing with subsistence living activities (37), such as farming and fishing, which promote consumption of traditional foods. In contrast, traditional food systems in high-income-economic-level jurisdictions have been disrupted by development through urbanization, tourism, and military expansion, reducing the availability of locally grown traditional foods and increasing the convenience of and reliance on imported and acculturated foods (38). Higher-income islands may be described as being at a later stage of the nutrition transition, whereas lower-middle-income islands are at an earlier stage of the nutrition transition, a concept outlined by Popkin (1, 2).

The islands of American Samoa, Palau, and RMI appear to be on the cusp of the nutrition transition due to being upper-middle-income economies, a level in-between the jurisdictions previously described (1, 2, 33). Children in American Samoa had a higher amount of F&V intake, with a relatively high amount of these F&Vs being traditional. At the other end of this economic level, Palau had a lower amount of F&V intake and less of the F&Vs consumed were traditional compared with American Samoa; however, Palau still had a slightly higher traditional intake than the high-income-economic-level jurisdictions. However, RMI had a low overall F&V intake, but about half of the F&Vs consumed were traditional. RMI has a unique topography of coral atolls; thus, the islands have limited land area and fewer freshwater sources for agriculture (39), which may be a reason for the overall lower consumption of F&Vs. However, possibly being at an earlier crossover stage of the nutrition transition could be why RMI children are consuming more traditional F&Vs.

Differences in consumption of traditional F&Vs may also be dependent on social norms and cultural values, which have been affected by increased development and westernization in the Pacific. Although all of the jurisdictions have been influenced by Western culture, the degree of influence varies by jurisdiction and is somewhat dependent on their affiliation with the United States. Hawaii, as a US state, is the most developed and westernized of all the jurisdictions. Guam, CNMI, and American Samoa are US territories, whereas Palau, RMI, and FSM are sovereign island nations having specialized relationships with the United States as FAS jurisdictions (32). Guam and CNMI have been more influenced by the United States as territories and having a longer history of colonization, which appears to have resulted in less practice of their traditional customs, much like Hawaii (40). This could further explain the lower intake of traditional foods among these jurisdictions compared with the FAS island nations. The culture and traditional practices in the FAS (Palau, RMI, and FSM) are strong today (41), which, along with factors associated with being lower-middle economies, may be encouraging the intake of traditional F&Vs among their children.

American Samoa seems to be unique, however, as their political status is a US territory, yet their children's consumption of traditional F&Vs is much higher, and even higher than in Palau (an FAS jurisdiction). Although American Samoa is a US territory similar to Guam and CNMI, they uphold much of their cultural values and exhibit traditional practices to a greater extent (41), which appear to include traditional foods, as shown by the results of this study. The people of Palau also practice traditional customs; however, their larger tourist industry may be introducing more outside influences to the local population (42), which is possibly another reason for their children's lower traditional F&V consumption. Similarly, Hawaii, Guam, and CNMI have large tourist industries, which may have indirectly influenced dietary practices as well.

Although modern capitalist worldviews have considered lower economic income levels, less development, and less association with Western societies as disadvantageous (43), this may potentially be beneficial in terms of promoting consumption of healthy traditional foods and sustaining the natural environment (1, 2, 13, 18, 44). Since their independence, the FAS islands have increased development, improved infrastructure, and introduced various industries for economic revenue, although at lower capacities than the US states and territories. Although this has initiated some nutrition transition in these islands, based on the results of this study, the FAS island nations, most notably FSM and RMI, appear to have an advantage over the other Pacific jurisdictions in preserving traditional food consumption given these transitory forces at a much earlier stage. The FAS islands’ stronger connection with their traditional ways of life are in their favor and can benefit their population's health if further encouraged and people are made aware of the advantages of their traditional lifestyles and diet (18, 22, 45, 46). Indeed, despite the outside influence of consumerism and capitalism forcing nontraditional foods into their primary diets, FSM still encourages traditional and local food consumption into their food systems and community through programs such as the “Go Local” food campaign and network, which promotes the production and consumption of traditional foods (18, 44, 47). The consumption of traditional F&Vs (and likely other traditional foods) in American Samoa, despite westernization, also demonstrates the preservation and perpetuation of traditional practices as a leverage point to adopting a healthy diet in the Pacific. For high-income Pacific economies, such as CNMI, Guam, and Hawaii, placing more value on and increasing traditional ways of living may assist in promoting the regulation of imported foods and support increased consumption of locally grown produce to advance the nutrition transition to desired societal/behavioral change (1, 4, 13, 19). These observations would support policies for cultural preservation, a self-sustaining local food system, and regulation of imports to achieve optimal food security.

There are several areas of interest that are beyond the scope of this study. This analysis evaluated the total recorded F&Vs within each jurisdiction and not by each child within a specific jurisdiction. Thus, these results are unable to enumerate whether the F&Vs consumed within jurisdictions were consumed equally across all children or if some children were consuming more F&Vs than others. Furthermore, it cannot be determined if the traditional F&Vs consumed were consumed by most of the children in the sample or if only a few children in the sample were consuming the majority of the traditional F&Vs. Quantifying F&V intake by each child within each jurisdiction may show that, although the total count of traditional F&Vs consumed in a specific jurisdiction is large, the number of children consuming traditional F&Vs may be small. Second, this study did not classify all F&Vs recorded as traditional or acculturated. This was due to the classification being based on an a priori list of F&Vs and only whole or single F&Vs being enumerated as traditional or acculturated. Third, language may have been a barrier in recording dietary records, although recording was done in the Indigenous language if preferred and then translated to English by bilingual CHL jurisdiction staff prior to data entry.

This study exclusively focused on F&Vs as one of the goals of the CHL program was to increase intake of these foods. As a result, only intakes of F&Vs were evaluated. Future studies would benefit from including the full spectrum of foods such as meats, seafood, or other protein groups, which are also shown to be highly consumed in the USAP region (4), as well as including a wider range of children's ages. Traditional protein sources of the Pacific generally include fish and other seafoods, and game meat from the land, which are both lower in fats. Acculturated and nontraditional modern meats may include processed or canned meats, which are much higher in fat and sodium. Assessing meat consumption in terms of traditional and acculturated would provide additional insight into diet and nutrition among children and adults in the Pacific. Future studies to assess traditional food intake among children using CHL data from Alaska would also be valuable. Although a Pacific jurisdiction, Alaska was not included in the current study due to the differences in climate and traditional foods from the tropical Pacific islands.

In summary, based on the results of this study, children of the USAP region as a whole would benefit from an increase in their overall intake of F&Vs. Likely due to increased development, convenience of imported foods limiting availability and access of traditional foods, and changes in cultural practices and social norms, high-income USAP jurisdictions consumed fewer traditional F&Vs compared with lower- and upper-middle-income jurisdictions where incorporation of traditional F&Vs into their children's diets are more common. Developing strategies to promote a greater frequency of consumption of all F&Vs, in particular traditional fruits and vegetables prepared by traditional practices, should benefit the health of people in the Pacific.

## Supplementary Material

nzac101_Supplemental_File

## Data Availability

Data described in the manuscript, code book, and analytic code will be made available upon request pending application at https://www.chl-pacific.org/chl-data/data-requests/, approval by the Children's Healthy Living program, and payment to cover any costs associated with the request.

## Notes

Supported by the Agriculture and Food Research Initiative grant 2011–68001-30335 from the US Department of Agriculture National Institute of Food and Agricultural Science Enhancement Coordinated Agricultural Program; the CHL Center of Excellence is currently funded by the USDA National Institute of Food and Agriculture, Agriculture, and Food Research Institute grant no. 2018-69001-27551, and the National Institutes of Health National Cancer Institute through grants P30 CA071789, 3P30CA071789-14S1 (internship for EW), and U54 CA143727 (fellowship for RDC).

Author disclosures: The authors report no conflicts of interest. The funders had no role in the design of the study; in the collection, analyses, or interpretation of data; in the writing of the manuscript, or in the decision to publish the results.

Supplemental Table 1 is available from the “Supplementary data” link in the online posting of the article and from the same link in the online table of contents at https://academic.oup.com/cdn/.

RDC and EW are co-first authors and contributed equally to this paper.

KAS is deceased.

Abbreviations used: CHL, Children's Healthy Living Program for Remote Underserved Minority Populations of the Pacific Region; CNMI, Commonwealth of the Northern Mariana Islands; FAS, Freely Associated States; FSM, Federated States of Micronesia; F&V, fruit and vegetable; GNI, gross national income; NCD, noncommunicable disease; PacTrac3, Pacific Tracker3; PICT, Pacific Island Country or Territory; RMI, Republic of the Marshall Islands; USAP, US-affiliated Pacific; USAPI, US-affiliated Pacific Islands.
