# Supplementary material for: Consumption of Traditional Fruits and Vegetables among Children in the US-Affiliated Pacific Region
Source: Curr Dev Nutr. 2022 Jun 16;6(7):nzac101. doi: 10.1093/cdn/nzac101 (PMC9283104; doi:10.1093/cdn/nzac101)
Supplement: nzac101_Supplemental_File [file nzac101_supplemental_file.docx]

**Consumption of Traditional Fruits and Vegetables Among Children in the U.S. Affiliated Pacific Region**

**Rica Dela Cruz et al.**

**Online Supplementary Material**

**Supplementary Table 1. Classification Form list of enumerated fruits and vegetables (n=126 food items)**

| Amaranth  Ambarella  Arrowroot  Avocado  Banana  Banana Sprout  Basil  Beans  Bell pepper  Betel nut  Bird's nest fern  Bitter gourd  Bittermelon  Bottle gourd, squash  Brazilian spinach  Breadfruit  Bush passion fruit  Cabbage  Calamansi  Canistel  Carrot  Cassava  Chaya  Chayote  Chestnut  Chili pepper  Chinese cabbage  Citrus fruit  Cocoa fruit  Coconut apple  Coconut cream  Coconut embryo | Coconut meat  Coconut milk  Coconut sprout  Coconut tuba or sap  Coconut water  Cucumber  Custard Apple  Dragon Fruit  Drumstick, moringa  Eggplant  False durian  Fern  Garlic pear  Garlic vine  Giant passion fruit  Giant swamp taro  Ginger  Golden apple  Grapefruit  Green Banana  Green Bean  Guava  Hibiscus  Indian almond  Indian mulberry  Jackfruit  Jambolan  Jungle apple  Kangkong, swamp cabbage  Kava  Kavika  Kumquat | Leafy green  Leek  Lemon  Lemon grass  Lettuce  Lime  Lychee  Mandarin  Mango  Mountain apple  Mountain Palm  Native cinnamon  Native fig  Nightshade  Noni  Oil palm  Okinawa spinach  Okra  Orange  Oriental radish  Palm  Panama Berry  Pandanus  Papaya  Passion fruit  Pepper  Pepper corn  Pineapple  Plantain  Pomelo  Potato | Prickly Pear  Pumpkin  Rambutan  Red bean  Rice  Rose apple  Sakau (drink)  Soft taro  Soursop  Spinach  Spring onion  Squash  Star apple  Starfruit  Sugar cane  Sunset hibiscus  Sweet Potato  Sweetleaf bush  Tahitian chestnut  Tamarind  Tangerine  Tapioca  Taro  Tomato  Turmeric  Water dropwort  Water Spinach  Watercress  Watermelon  Winged bean  Yam |
| --- | --- | --- | --- |
